# Consenso internacional sobre nomenclatura en equinococosis: traducción y adaptación al español

**DOI:** 10.17843/rpmesp.2024.412.13589

**Published:** 2024-06-11

**Authors:** Ariel Naidich, María Celina Elissondo, Katherina Vizcaychipi, Guzman Sienra, Viterman Ali, Cesar M. Gavidia, Jorge Guisantes

**Affiliations:** 1 Departamento de Parasitología, Instituto Nacional de Enfermedades Infecciosas, ANLIS “Dr. Carlos G. Malbrán”. Buenos Aires, Argentina. Departamento de Parasitología Instituto Nacional de Enfermedades Infecciosas, ANLIS “Dr. Carlos G. Malbrán” Buenos Aires Argentina; 2 Instituto de Investigaciones en Producción Sanidad y Ambiente (IIPROSAM CONICET-UNMdP), Facultad de Ciencias Exactas y Naturales - UNMdP, Centro Científico Tecnológico Mar del Plata (CONICET), Centro de Asociación Simple CIC PBA, Mar del Plata, Argentina. Instituto de Investigaciones en Producción Sanidad y Ambiente (IIPROSAM CONICET-UNMdP) Facultad de Ciencias Exactas y Naturales - UNMdP Centro Científico Tecnológico Mar del Plata (CONICET), Centro de Asociación Simple CIC PBA Mar del Plata Argentina; 3 Instituto Nacional de Medicina Tropical ANLIS “Dr. Carlos G. Malbrán”, Puerto Iguazú, Misiones, Argentina. Instituto Nacional de Medicina Tropical ANLIS “Dr. Carlos G. Malbrán” Puerto Iguazú Misiones Argentina; 4 Instituto de Investigación en Veterinaria, Facultad de Ciencias Agrarias y Veterinarias, Universidad del Salvador, Corrientes, Argentina. Universidad del Salvador Instituto de Investigación en Veterinaria Facultad de Ciencias Agrarias y Veterinarias Universidad del Salvador Corrientes Argentina; 5 Programa Nacional de Control de Zoonosis y Centro Antirrábico Nacional, Ministerio de Salud Pública y Bienestar Social, Asunción, Paraguay. Programa Nacional de Control de Zoonosis y Centro Antirrábico Nacional Ministerio de Salud Pública y Bienestar Social Asunción Paraguay; 6 Instituto de Investigación en Salud y Desarrollo (IINSAD), Cátedra de Parasitología, Facultad de Medicina, Universidad Mayor de San Andrés (UMSA), La Paz, Bolivia. Universidad Mayor de San Andrés Instituto de Investigación en Salud y Desarrollo (IINSAD), Cátedra de Parasitología Facultad de Medicina Universidad Mayor de San Andrés (UMSA) La Paz Bolivia; 7 Facultad de Medicina Veterinaria, Universidad Nacional Mayor de San Marcos, Lima, Perú. Universidad Nacional Mayor de San Marcos Facultad de Medicina Veterinaria Universidad Nacional Mayor de San Marcos Lima Peru; 8 Departamento de Inmunología, Microbiología y Parasitología, Universidad del País Vasco, Vitoria, España. Universidad del País Vasco Departamento de Inmunología, Microbiología y Parasitología Universidad del País Vasco Vitoria Spain

**Keywords:** Reuniones de consenso como asunto, terminología, equinococosis, Echinococcus

## Abstract

La equinococosis se conoce desde tiempos de Hipócrates. Desde entonces se acuñaron o adaptaron términos y definiciones en diferentes idiomas para referirse a múltiples aspectos relacionados con esta zoonosis. Es importante propiciar un buen entendimiento en la lectura y escritura en la información científica, académica, para el conocimiento general y la difusión masiva. Durante el 27º Congreso Mundial de Equinococosis se estableció un grupo de trabajo con ese propósito. El resultado fue un consenso con recomendaciones aplicables a la ciencia y comunicación entre profesionales, publicado en el año 2020 en idioma inglés. Por recomendación de miembros del Grupo de Trabajo Informal de la OMS sobre equinococosis (WHO-IWGE) se convocó a un grupo de trabajo integrado por expertos en equinococosis de Argentina, Bolivia, Chile, España, Paraguay, Perú y Uruguay para elaborar una propuesta de terminología en español. Este consenso propone una nomenclatura unificada de aplicación progresiva, que sirve como base de consulta y referencia.

## INTRODUCCIÓN

Los parásitos del género *Echinococcus* pertenecen a la clase Cestoda dentro del filo *Platyhelminthes*. Se reconocen nueve especies dentro del género que, desde el punto de vista etiológico, clínico, patológico y epidemiológico, producen tres grupos de enfermedades zoonóticas: la equinococosis quística (EQ), la equinococosis alvelolar (EA) y la equinococosis neotropical (EN).

La importancia de las equinococosis como zoonosis parasitarias de interés humano y animal, llevó a la creación de la Asociación Internacional de Hidatidología en 1941, en Colonia del Sacramento (Uruguay) a fin de difundir la relevancia de dicha zoonosis (especialmente la EQ) en los países endémicos, potenciar su control y recabar el interés de las autoridades sanitarias. Inicialmente el idioma oficial fue el español, pero a partir de 1951 y hasta finales de los 90, el inglés y el francés se incorporaron como idiomas oficiales para sus congresos bianuales, convocados también bajo la denominación de *International Association of Hydatidology* (IAH).

En el XXVI congreso de la IAH realizado en Bucarest, Rumania, en el año 2015, el nombre oficial en inglés de la asociación pasó a ser *World Association of Echinococcosis* (WAE), englobando así las diferentes enfermedades producidas por las especies del género *Echinococcus*. En 1985 fue creado, dentro de la Organización Mundial de la Salud (OMS/WHO), el grupo *WHO Informal Working Group on Echinococcosis* (IWGE) con el doble objetivo de formar una red de científicos trabajando en ciencias básicas en el campo de la equinococosis, así como la estandarización de métodos para el diagnóstico y tratamiento de las enfermedades producidas por *Echinococcus* spp. Ambas entidades (WAE e IWGE) tienen sus principales reuniones en las mismas ciudades, uniendo así sus esfuerzos para la edición de manuales sobre equinococosis que incluyen aspectos de prevención, vigilancia y control bajo el enfoque de Una Salud, así como para actualizar guías para el tratamiento y diagnóstico de pacientes con equinococosis.

La diversidad de enfermedades producidas y su extensión por diferentes países y continentes dio lugar, a través de la evolución histórica de la enfermedad, a numerosos términos para designar tanto las formas parasitarias, así como las presentaciones y cuadros clínicos.

Es así como en la literatura científica, profesional y legal, figuran diferentes términos para la EQ, tales como hidatidosis, enfermedad hidatídica, quiste hidatídico, equinococosis cística; para la EA se encuentran los términos como alveococosis, *Echinococcus alveolaris*, hidatidosis alveolar, enfermedad hidatídica alveolar, quistes multiloculares hidatídicos y enfermedad hidática multilocular; finalmente, para la EN encontramos nombres como equinococosis poliquística, enfermedad hidatídica poliquística, hidatidosis del Nuevo Mundo, etc.

En una sesión plenaria en el *XXVII World Congress of Echinococcosi*s (Argel, 2017) se hizo hincapié sobre la necesidad de armonizar la terminología sobre equinococosis basándose en argumentos científicos y lingüísticos sólidos. Eso dio lugar a un grupo de trabajo y consenso que se describe en Vuitton *et al*. ^1^ donde asimismo se detallan los resultados consensuados. En dicho artículo también se propuso que las recomendaciones recogidas en dicha publicación pudieran ser la base para su aplicación en otros idiomas. Eso ha dado lugar a la publicación de una versión francesa ^2^ y china ^3^ de terminología recomendada en equinococosis.

Para el consenso en dicha terminología se tuvo en cuenta la denominación binomial de géneros y especies de acuerdo al *International Code of Zoological Nomenclature* (ICZN) ^4^. Asimismo, se siguió *la Standardized Nomenclature of Parasitic Diseases* (SNOPAD) adoptada por la *World Federation of Parasitologists* de acuerdo a una propuesta anterior de la *World Association for the Advancement of Veterinary Parasitologists* en 1988 ^5^. Entre las recomendaciones de dichas nomenclaturas, está la de denominar las enfermedades parasitarias de acuerdo al género del parásito seguido por el sufijo “osis” (por ejemplo, de *Echinococcus* daría lugar a echinococcosis). La aplicación de esta regla no siempre se ha seguido y coexiste con nombres prevalentes especialmente en el área de la medicina humana y veterinaria.

Un ejemplo es el idioma español, donde el término aceptado por la Real Academia Española es “equinococosis”, que figura asimismo en diccionarios médicos ^6^, siendo éste utilizado en la literatura en español, tanto científica como en legislación, incluyendo las revistas y publicaciones de la Organización Panamericana de la Salud (OPS).

El Grupo de Trabajo Informal de la OMS sobre equinococosis (WHO Informal Working Group on Echinococcosis) convocó a un grupo de trabajo integrado por expertos en equinococosis de (en orden alfabético) Argentina, Bolivia, España, Paraguay, Perú y Uruguay, grupo coordinado por Ariel Naidich (Argentina) para elaborar una propuesta de terminología de equinococosis en español, dada la importancia de dichas parasitosis como enfermedades endémicas en países de habla española. Se realizaron consultas específicas con profesionales de Brasil y Chile. El objetivo de la presente publicación es presentar dicha propuesta para armonizar la terminología en este idioma.

## METODOLOGÍA

Inicialmente, los miembros del equipo de trabajo tradujeron las tablas del consenso en idioma inglés ^1^ de forma literal. Cada término o expresión fue sometido a lectura conjunta (en reuniones virtuales) donde de acuerdo a cada país o región, se compartió el significado, utilización, posibles confusiones, validez lingüística, etc. Para los términos que resultaron conflictivos o presentaban dudas se consultaron fuentes lingüísticas y de usos y costumbres en los diferentes países, como así también la legislación y documentación publicada en el marco de difusión y programas de control ^6^^-^^8^. En estos casos se consensuó entre los miembros del equipo la elección de una expresión única, agregándose en los argumentos las particularidades según fuese necesario de acuerdo al aporte, conocimiento y experiencia de los representantes de cada país. Los resultados fueron ingresados a las tablas de acuerdo al agrupamiento temático de los mismos. Para la traducción de los términos incluidos en las figuras se procedió de igual manera que con las tablas. Las tablas se revisaron nuevamente antes de su envío a publicación para verificar la coherencia interna.

**Figura 1:**
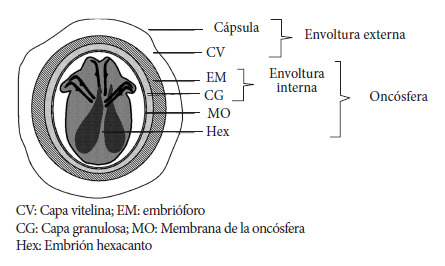
Terminología descriptiva en español del huevo resultante de la reproducción sexual de parásitos del género *Echinococcus*. (Adaptado con autorización de Vuitton DA *et al*. Parasite. 2020;27:41).

**Figura 2:**
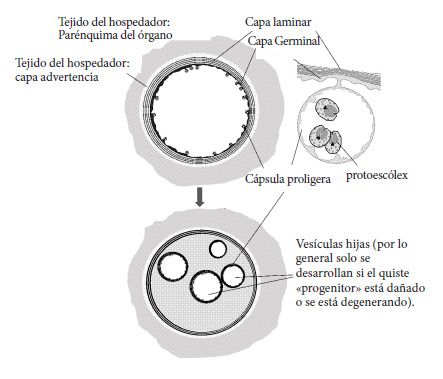
Terminología descriptiva en español del metacestodo (forma larvaria, “quiste hidatídico”) de *Echinococcus granulosus sensu lato*: ejemplo de localización hepática (Adaptado con autorización de Vuitton DA et al. Parasite. 2020;27:41).

**Figura 3:**
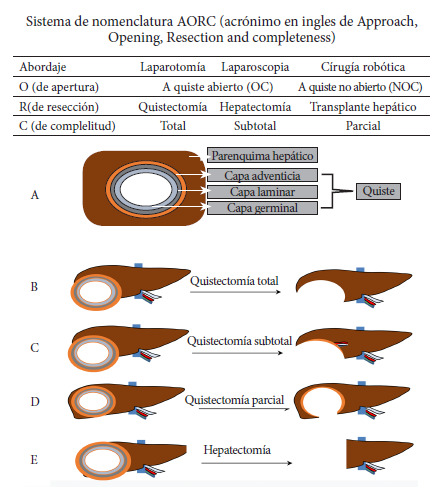
Sistema AORC (Acrónimo en inglés correspondiente a Abordaje-Apertura-Resección-Completitud) para la descripción de intervenciones quirúrgicas para el tratamiento de la equinococosis quística (EQ) de localización hepática. Equivalentes españoles de la terminología internacional (Adaptado con autorización de Vuitton DA *et al*. Parasite. 2020;27:41).

Los términos en español recomendados para la genética y epidemiología de las especies de *Echinococcus* se presentan en la Tabla 1, los términos recomendados en español para la biología e inmunología de especies del género *Echinococcus* en la Tabla 2, los términos en español recomendados para aspectos clínicos de las equinococosis en la Tabla 3 y los términos en español rechazados para la genética, la biología e inmunología de especies de *Echinococcus* y la clínica, se muestran en la Tabla 4, Tabla 5 y Tabla 6. Asimismo, en la Figura 1 se muestra la terminología descriptiva en español del huevo resultante de la reproducción sexual de parásitos del género *Echinococcus,* en la Figura 2 la terminología descriptiva en español del metacestodo y en la Figura 3 el Sistema AORC (Acrónimo en inglés correspondiente a Abordaje-Apertura-Resección-Completitud) para la descripción de intervenciones quirúrgicas para el tratamiento de la equinococosis quística (EQ) de localización hepática ^1^. Las referencias bibliográficas en el presente trabajo no incluyen todas las referencias del trabajo del consenso en idioma inglés.

**Tabla 1:** Términos en español recomendados para la genética y epidemiología de las especies de *Echinococcus*.

Palabra/ expresión	Definición	Argumentos para aceptar este término, referencias, aclaraciones lingüísticas, comentarios
*Echinococcus* Rudolphi, 1801 (Cestoda: Taeniidae)	Género de la familia Taeniidae Ludwig, 1886, orden *Cyclophyllidea*, clase *Cestoda*, *phylum Platelmintos*, Reino Animal.	Nomenclatura internacional: nombre del género *Echinococcus*. Debe estar escrito en cursiva con la primera letra en mayúscula; abreviatura “*E*” (cursiva seguida de un punto) cuando forme un binomen (es decir, un nombre genérico y un nombre específico) o en un trinomen (un nombre genérico, un nombre específico y un nombre subespecífico). El nombre del género Alveococcus (para la especie *Alveococcus multilocularis;* Leuckart, 1863; Abuladze, 1959) fue adoptado para separar *E. multilocularis* de las otras especies. Ya no hay una base taxonómica para tal separación, pero el nombre todavía se usa ocasionalmente, particularmente en la literatura rusa. Consulte también la Tabla 4.
*Echinococcus* sp.	Una especie dentro del género *Echinococcus*.	La abreviatura “sp.” (no cursiva) representa una especie cuya identidad no se conoce (por ejemplo, en caso de aislamientos de *Echinococcus* indeterminados).
*Echinococcus* spp.	Más de una especie (o todas) dentro del género *Echinococcus*.	La abreviatura “spp.” no indica una especie con la definición taxonómica de “especie”, pero significa “species pluralis” (el latín para “múltiples especies”), aquí en la letra romana (no cursiva) spp va seguido de un punto spp. (porque es la abreviatura de una expresión latina).
*Echinococcus canadensis* (Webster & Cameron, 1961)	Una especie dentro del complejo de especies del *E. granulosus sensu lato*	*E. canadensis* (Webster y Cameron, 1961) corresponde a los genotipos “G6/G7”, “G8” y “G10” identificados por secuenciación de ADN; *E. canadensis* pertenece a *E. granulosus s.l.* Los genotipos pueden usarse para diferenciar entre distintas secuencias moleculares dentro de la especie definida. La palabra “cepa” ya no debe utilizarse. El ciclo de vida del *E. canadensis* puede involucrar caprinos y camélidos (G6), porcinos (G7) y cérvidos (G8 y G10) como hospedadores intermediarios y cánidos domésticos (perros) y silvestres (lobos para G8 y G10) como hospedadores definitivos; sin embargo, ovinos, bovinos y otros ungulados también pueden estar infectados por *E. canadensis* que es la segunda especie en importancia en infecciones humanas dentro de *E. granulosus s.l*., después de *E. granulosus s.s*. Los genotipos G6 y G7 podrían separarse en el futuro de esta especie, por lo que en la actualidad se recomienda la expresión “Grupo *E. canadensis*”.
*Echinococcus equinus* (Williams & Sweatman, 1963)	Especie del complejo *E. granulosus s.l.*	*E. equinus* (Williams y Sweatman, 1963) corresponde al genotipo “G4”, identificado por secuenciación de ADN. *E. equinus* pertenece a *E. granulosus s.l*. El ciclo de *E. equinus* generalmente involucra a miembros de la familia de los équidos como hospedadores intermediarios y a los cánidos domésticos como hospedadores definitivos. También se conoce un ciclo de vida silvestre que involucra leones y cebras. Se asocia con enfermedades en primates no humanos malgaches (lémures). Sin embargo, *E. equinus* presenta un potencial zoonótico aún no demostrado de manera convincente.
*Echinococcus felidis* (Ortlepp, 1937)	Especie del complejo *E. granulosus s.l*.	*E. felidis* (Ortlepp, 1937) fue descrito por primera vez como tal en león, *Panthera leo*, en Sudáfrica. En la actualidad es reconocida como una especie distinta, identificada por secuenciación de ADN; *E. felidis* pertenece a *E. granulosus s.l*. El ciclo de *E. felidis* involucra a los leones como hospedadores definitivos y sólo se conoce a los jabalíes y a los hipopótamos como hospedadores intermediarios. A la fecha de publicación del presente trabajo, no se ha reconocido que *E. felidis* esté asociado a infecciones en humanos.
*Echinococcus granulosus* (Batsch, 1786) *sensu lato*	El concepto de *E. granulosus* como un grupo de especies que contiene todos los agentes de la equinococosis quística.	*“sensu lato”* se utiliza en un sentido más amplio para el nombre de una especie (aquí: *E. granulosus* (Batsch, 1786)) que incluye, por ejemplo, especies crípticas. *“sensu lato”* debe estar en cursiva (aunque esto es debatido), sin mayúsculas en las primeras letras. “*E. granulosus*” debe ir seguido de “*sensu lato*” siempre que no se haya determinado la especie precisa. La abreviatura de cada palabra en “*sensu lato*” va seguida de un punto (porque es la abreviatura de las palabras latinas), así: “*s.l.*” Para publicaciones sobre los aspectos clínicos de la equinococosis, dado que las descripciones clínicas de la enfermedad “equinococosis quística” (EQ) encaja con todas aquellas especies de *E. granulosus s.l*. que son responsables de enfermedades en humanos, el uso de *E. granulosus s.l.* está permitido cuando no hay identificación de la especie real. Para las publicaciones sobre investigaciones básica y epidemiología es necesaria la identificación molecular de la especie real; debe realizarse e indicarse en la sección de Materiales y Métodos.
*Echinococcus granulosus* (Batsch, 1786) *sensu stricto*	Especie del complejo *E. granulosus s.l.*	*“sensu stricto”* y *“sensu lato”* se utilizan cuando el nombre de una especie (aquí: *E. granulosus* (Batsch, 1786)) se usa en diferentes conceptos: en un sentido más amplio (*s.l.*) que incluye especies crípticas, o en un concepto más restringido (*s.s.*). “*sensu stricto*”, que es el equivalente de una especie, debe estar en cursiva (aunque esto se debate), sin mayúsculas como primeras letras. La abreviatura de cada palabra en “*sensu stricto*” va seguida de un punto (porque son las abreviaturas de las palabras latinas), así: “*s.s.*”. *E. granulosus* *s.s*. pertenece a *E. granulosus s.l*. *E. granulosus s.s.* corresponde a los genotipos “G1”, “G2”, “G3” y una microvariante del genotipo “G3”, identificados por secuenciación de ADN; su ciclo de vida suele incluir ovinos como hospedadores intermediarios y perros como hospedadores definitivos; los bovinos y otros ungulados también pueden ser infectados por *E. granulosus s.s*. El genotipo “G Omo”, una variante genética divergente de África se retiene provisionalmente en *E. granulosus s.s.*, pero tendrá que ser reclasificado en el futuro.
*Echinococcus multilocularis* (Leuckart, 1863)	Especie del género *Echinococcus.*	*E. multilocularis* (Leuckart, 1863) es el agente de la “equinococosis alveolar” (EA) en humanos y se distinguió claramente de *E. granulosus s.l.* a mediados del siglo XX. Sus hospedadores intermediarios incluyen una variedad de roedores y lagomorfos dependiendo del área geográfica, siendo los humanos hospedadores intermediarios accidentales y varias especies de zorros, perros y lobos como hospedadores definitivos. La enfermedad causada por *E. multilocularis*, caracterizada por lesiones compuestas por un agregado de microquistes, incrustados en la reacción granulomatosa del hospedador, es distinta de la causada por *E. granulosus s.l*. así como las causadas por *E. vogeli* y *E. oligarthra*. Desde entonces, no se ha encontrado ningún polimorfismo genético importante dentro de *E. multilocularis* que distinga nuevas especies o variantes genéticas.
*Echinococcus oligarthra* (Diesing, 1863)	Especie del género *Echinococcus*.	*E. oligarthra* (Diesing, 1863) es una especie que se encuentra en América del Sur, Central y del Norte (México). El componente “arthra”, propuesto originalmente por Diesing, proviene del griego antiguo; αρθρα-arthra (articulaciones) que es el plural de αρθρ*o*ν-arthron (articulación). Por tanto, el nombre no es un adjetivo, sino un sustantivo en aposición, que no cambia su terminación según el género del nombre genérico. Esto se reconoció antes, pero posteriormente se ignoró. El ciclo de *E. oligarthra* involucra principalmente a agutíes (*Dasyprocta spp.*) y ocasionalmente a pacas (*Cuniculus paca*), ratas espinosas (*Proechimys spp.*), conejos (*Sylvilagus floridanus*) y zarigüeyas (*Didelphis marsupialis*) como hospedadores intermediarios y a felinos domésticos y silvestres como hospedadores definitivos. En humanos produce una enfermedad distinta a la equinococosis quística y alveolar, a veces denominada erróneamente “equinococosis poliquística” (por su apariencia macroscópica similar al metacestodo de *E. vogeli* pero *E. oligarthra* se presenta como un quiste o vesícula, único o agrupado). Ver también *E. vogeli* Tabla 1 y Tabla 3.
*E. ortleppi* (Lopez-Neyra & Soler Planas, 1943)	Especie del complejo *E. granulosus s.l.*	*E. ortleppi* (Lopez-Neyra y Soler Planas, 1943) corresponde al genotipo “G5”, identificado por secuenciación de ADN. El ciclo de *E. ortleppi* generalmente involucra a bovinos como hospedadores intermediarios y a los perros como hospedadores definitivos; otros ungulados también pueden estar infectados por *E. ortleppi*. Se conocen casos humanos, pero son poco frecuentes o no han sido publicados.
*E. shiquicus* (Xiao, Qiu, Nakao, Li, Yang, Chen, Schantz, Craig & Ito, 2005)	Especie del género *Echinococcus.*	*E. shiquicus*^9^ es una especie filogenéticamente cercana a *E. multilocularis* que fue identificada en el condado de Sêrxü en la provincia de Sichuan, región occidental de la meseta Qinghai-Tíbet de la República Popular China. La infección por *E. shiquicus* no ha sido reportada en humanos a la fecha.
*E. vogeli* (Rausch & Bernstein, 1972)	Especie del género *Echinococcus*.	*E. vogeli*^10^ es una especie de *Echinococcus* que se encuentra sólo en América del Sur y Central donde se encuentran presentes sus hospedadores silvestres. El ciclo natural de *E. vogeli* involucra como hospedador definitivo al zorro pitoco (*Speothos venaticus*) y como hospedador intermediario principalmente a paca (*Cuniculus paca*); también se ha documentado en otros roedores como el agutí (*Dasyprocta* spp.), rata espinosa (*Proechimys* spp.) y armadillo (*Dasypus novemcinctus*). El perro doméstico también se comporta como hospedador definitivo. *E. vogeli* es responsable de una enfermedad en humanos distinta de la equinococosis quística y alveolar, a menudo denominada “equinococosis poliquística”, debido a la presentación de la enfermedad. “Equinococosis neotropical poliquística” es la expresión recomendada para calificar la infección humana debida a *E. vogeli*: ver también la Tabla 3.
Genotipos (G)	Genotipos identificados dentro de la especie *E. granulosus s.l*., previamente denominados como “cepas”.	Los genotipos pueden usarse para diferenciar entre distintas secuencias moleculares, dentro de las especies o complejos definidos; la palabra “cepa” ya no debe utilizarse cuando se haya realizado la caracterización genética. Aún es posible una mayor definición de nuevas especies. A la espera de tales definiciones, los genotipos deberían mantenerse, además del nombre actual de la especie, si se dispone de la información.
Genotipos G1, G2 y G3	Genotipos individualizados dentro de *E. granulosus s.s*.	Mantener la distinción entre genotipos puede ser necesario para los estudios filogeográficos y, en algunas circunstancias, para el control de la equinococosis (dirigidos a hospedadores animales y ciclos específicos). G1 es el genotipo más común en ovinos. G2 se describió por primera vez como “cepa de oveja de Tasmania”, pero en realidad es cosmopolita; G3 se identificó inicialmente en búfalos de agua, pero ahora también se conoce en otros hospedadores intermediarios. G2 se considera una microvariante de G3.
Genotipos G6/G7, G8 y G10	Genotipos individualizados dentro de *E. canadensis* (Webster y Cameron, 1961).	A la espera de una mayor distinción entre las especies aceptadas, los genotipos de *E. Canadensis* deben calificarse con la nomenclatura previamente aceptada: G6/G7, G8 y G10. G9 ya no se reconoce como un genotipo distinto; probablemente sea una microvariante de G7. El grupo genotípico G6/G7 es el segundo agente más importante de la EQ humana en todo el mundo. Se ha propuesto una mayor distinción entre especies dentro del grupo de *E. canadensis,* pero aún no se ha aceptado.

Adaptado con autorización de Vuitton DA *et al.* Parasite. 2020;27:41

**Tabla 2:** Términos recomendados en español para la biología e inmunología de especies del género *Echinococcus.*

Palabra/ expresión	Definición	Argumentos para aceptar este término, referencias, aclaraciones lingüísticas, comentarios
Abortado (del inglés aborted). Aplicable al quiste o lesión, adjetivo	Estructura parasitaria (hidátide) no viable según imagen (por calcificación completa en EA o imagen Tipo CE5 en EQ), y/o ausencia de células parasitarias viables en estudio histológico, trasplante o cultivo.	El adjetivo “abortado” no se utiliza en español en situaciones clínicas en la EQ humana, ni se encuentra habitualmente en la literatura científica en dicho idioma. Sí aparece en la nomenclatura inglesa ^(1)^. Se consideró de utilidad mencionarlo en esta nomenclatura en español a los efectos de facilitar la comprensión de un eventual lector que encontrase dicha denominación en un texto traducido del inglés.
Inactivo (quiste), adjetivo	Estructura parasitaria no activa, como se evidencia por imágenes (calcificación completa en quistes EA y EQ5).	Puede usarse en situaciones experimentales cuando se evidencia ausencia de viabilidad *in vivo* o *in vitro*. En situaciones clínicas esta denominación solamente se refiere a una evaluación biológica del parásito, pero que no siempre indica ausencia de enfermedad (debida por ejemplo en la equinococosis hepática, a una patología relacionada con la capa adventicia y comunicación biliar).
Borde periparasítico, expresión	Para *E. granulosus s.l.*, *E. multilocularis* y *E. vogeli,* estructuras que rodean la capa laminar de la hidátide, en el espacio virtual entre el parénquima del órgano y el quiste.	Término alternativo a “espacio virtual”.
Capa adventicia, expresión	Capa celular y fibrosa originada p or el hospedador intermediario que rodea las 2 capas interiores de origen parasitario (capas laminar y germinal) del metacestodo de *E. granulosus s.l.*, *E. vogeli* y *E. oligarthra*.	La descripción correcta de las estructuras parasitarias en hospedadores intermediarios incluye así 3 expresiones, (ver figura 2).
Capa germinal, expresión	La capa más interna del metacestodo de *Echinococcus* spp. que incluye varios tipos de células y produce otros componentes biológicos del metacestodo (líquido hidatídico, cápsulas prolígeras y protoescólex).	El término “capa” se prefiere al de “membrana germinal” debido a la complejidad de varias “capas” de la hidátide y la posible confusión del término membrana con la membrana celular, que tiene una definición específica en Biología. La descripción correcta de estructuras parasitarias en el hospedador intermediario incluye 3 expresiones (Figura 2).
Capa laminar, expresión	Parte exterior periférica acelular del metacestodo de *Echinococcus* spp., mayormente compuestos por mucopolisacáridos.	La capa laminar es producida por el parásito y ejerce funciones importantes en la interacción entre el metacestodo y el hospedador intermediario. El término “capa” debe usarse de preferencia debido a la complejidad de varias “capas” de la hidátide y la confusión posible de “membrana” con membrana celular, que tiene definición específica en biología. La descripción correcta de estructuras parasitarias en el hospedador intermediario incluye 3 expresiones (Figura 2).
Cápsula prolígera, expresión	Elementos que brotan de la capa germinal en donde se producen los protoescólex.	En algunas publicaciones se utiliza “vesícula prolígera”, sugiriéndose que se utilice solamente el término “cápsula prolígera”. No confundir con “vesículas hijas”. Las dos entidades son totalmente diferentes en su origen y sus componentes (ver Figura 2).
Célula germinativa, expresión	Célula madre somática pluripotente. Las células germinativas son las únicas mitóticamente activas en el metacestodo y dan origen a todas las células diferenciadas.	Es la expresión más comúnmente utilizada en la literatura, con esta definición. Sin embargo, debe notarse que no todas las células de la capa germinal son células germinativas.
Equinococósico, adjetivo	Adjetivo propuesto en el consenso en idioma inglés para calificar cualquier cosa relacionada a las especies de *Echinococcus* spp, sin uso en idioma español.	Término totalmente genérico que no presume sobre especies o etapas en las especies de *Echinococcus*. Basado en las recomendaciones de la Federación Mundial de Parasitología de acuerdo a los nombres de enfermedades parasitarias.
Escólex (plural: escólex), sustantivo	Primer segmento de la forma adulta de cestodos.	Del griego antiguo (“scolex”, genitivo “scolecos” y no “escolicos”). En el uso coloquial y definiciones antiguas corresponde a la “cabeza” en el esquema “cabeza-cuello y cuerpo” para cestodos. También se utiliza en el caso de protoescólex para la porción anterior que incluye los ganchos y ventosas.
Fértil (relativo a la forma adulta del cestodo), adjetivo	Presencia de huevos (infectivos para el hospedador intermediario) dentro del último proglótide de la forma adulta de *Echinococcus* spp. en el hospedador definitivo.	La producción de huevos observables en el último segmento de la forma adulta infiere que el helminto es fértil (por el contrario, la ausencia de huevos no permite inferir automáticamente que no es fértil, puede solamente ser inmaduro).
Fértil (metacestodo, larva, quiste, microquiste), adjetivo	Estructura parasitaria larvaria que contiene protoescólex viables y por lo tanto permite infección de hospedador definitivo a través de los mismos o en algunas circunstancias patológicas o experimentales, producción de metacestodos desarrollados en el hospedador intermediario.	Si existe producción de protoescólex por la capa germinal y su liberación en el líquido hidatídico (sea cual sea la especie) se infiere que el metacestodo es fértil.
Forma adulta, expresión	Etapa de reproducción sexual de parásitos de *Echinococcus* spp. en hospedadores definitivos.	No hay palabra alternativa científica para designar esta etapa en el desarrollo de cestodos *Echinococcus* spp. En oposición a la “forma larvaria” en hospedadores intermediario, “adulto” puede incluir todas las etapas del desarrollo en el hospedador definitivo (gusanos fértiles o inmaduros, es decir con huevos en el último proglótide o sin ellos).
Forma larvaria (o larval), expresión	Etapa de proliferación asexual de parásitos *Echinococcus* spp. en el hospedador intermediario.	La expresión puede ser usada como equivalente de metacestodo (o larva) para comunicar al público general aunque se prefiere “metacestodo” para comunicaciones científicas.
Ganchos, sustantivo	Apéndices de la forma adulta de parásitos de *Echinococcus* spp. que le permiten fijarse a la pared intestinal de hospedadores definitivos.	Este término debe ser usado tanto para los ganchos de la forma adulta como para los ganchos rostelares de los protoescólex.
Gusano, sustantivo	Etapa estrobilar de *Echinococcus* spp. en el hospedador definitivo.	Se utiliza como un equivalente muy popular de “forma adulta”, aunque su uso es desaconsejado en el lenguaje académico por prestarse a confusión con otros invertebrados.
Hidátide, sustantivo	Descripción parasitológica de la forma larval de cestodos tipo quiste asexual; más específicamente la descripción de la última etapa del metacestodo de *E. granulosus s.l*. y *E. vogeli.*	Según usos y costumbres del lenguaje, utilizado para diferenciar la hidátide del quiste hidatídico (por la presencia de capa adventicia).
Hidatídico, adjetivo	Se refiere al metacestodo de *Echinococcus* spp. en el hospedador intermediario, más específicamente se refiere al metacestodo de *Echinococcus granulosus s.l.*	Del griego antiguo:-hydatis-, genitivo -hydatidos (vesicular/vejiga llena de agua), hidatídico, que describe el estadío larval (metacestodo) de *Echinococcus* spp. nunca debe ser usado para la etapa adulta.
Huevo, sustantivo	Producto de la fecundación hermafrodita en la última proglótide de la forma adulta de parásitos del género *Echinococcus* spp. liberados al ambiente en las heces del hospedador definitivo. Es la forma infectante del parásito.	Debe ser restringido a las etapas previas a la ingestión por el hospedador intermediario y la liberación de oncósferas. Ver figura 1 para descripción detallada y terminología.
Infiltrado (periparasitario), sustantivo	Componentes histológicos (celulares y fibrosos) de origen en el hospedador en equinococosis alveolar; en contrario de la “capa adventicia” en quistes de EQ, los infiltrados celulares en EA no tienen límites claros con el parénquima hepático adyacente.	Término alternativo a “infiltración”.
Inmaduro (forma adulta), adjetivo	La forma adulta de *Echinococcus* spp. en el hospedador definitivo cuando el ultimo proglótide no contiene huevos (es decir al menos temporalmente no infeccioso para hospedadores intermediarios)	“Inmaduro” justamente indica que la forma adulta no ha desarrollado completamente a la etapa de producción de huevos; no se infiere que el parásito adulto no será fértil nunca, como puede sugerir la alternativa “no fértil”.
Líquido hidatídico, expresión	Líquido secretado por la capa germinal del metacestodo de *Echinococcus* spp. colectado en el centro del quiste	Debe reservarse para situaciones *in vivo,* quistes hidatídicos en EQ y microquistes en EA, EN para hospedadores intermediarios humanos y animales, incluyendo modelos experimentales.
Líquido vesicular, expresión	Líquido producido *in vitro* por el metacestodo de *Echinococcus* spp. en cultivo, cualquiera que sea la especie.	Este término debe reservarse para situaciones *in vitro* en las que se acordó usar el término “vesícula”.
Metacestodo (singular) metacestodos (plural), sustantivo	Forma de reproducción asexual de *Echinococcus* spp. en los hospedadores intermediarios; es la segunda fase de desarrollo que incluye todos los estadios, desde el post-oncosférico hasta el fértil (con producción de protoscólex, si los hay).	Designación científica para estas formas en parásitos cestodos en los hospedadores intermediarios; larva es un nombre alternativo para comunicar al público general. El término metacestode es equivalente. En el presente trabajo utilizamos metacestodo y se sugiere esa forma de uso.
Microquístes (equinococósico, *E. multilocularis*, EA), sustantivo microquístico, adjetivo	Múltiples quistes pequeños (de menos de 1 cm de diámetro) con capa germinal, capa laminar e infiltrado periparasitario de células del hospedador y fibrosis, lesiones características de la EA (debida a *E. multilocularis*).	Debe reservarse para lesiones de EA *in vivo;* puede observarse con técnicas de imágenes específicas, como la resonancia magnética nuclear (RMN) (imágenes ponderadas en T2) en humanos o modelos preclínicos, y/o en exámenes histológicos en modelos experimentales. Los microquistes son estructuras parasitarias, distintas de la cavidad necrótica central que se desarrolla a menudo en las lesiones de EA (“pseudoquiste” debe utilizarse para esta cavidad). Véase también la entrada “pseudoquiste” en esta tabla. En esta situación, “micro” no se refiere a “microscópico” sino a pequeñas formas macroscópicas.
No fértil (metacestodo, larva, quiste, microquiste, protoescólex), adjetivo	Estructura del parásito presente en el hospedador intermediario que no contiene protoescólex viables y, por lo tanto, es incapaz de infectar al hospedador definitivo.	Un metacestodo “no fértil” puede ser “viable”, estos adjetivos no son sinónimos.
No viable (aplicable a metacestodo, larva, quiste, microquiste, protoescólex), adjetivo	Estructura parasitaria que en un hospedador intermediario no contiene células vivas capaces de proliferar en condiciones adecuadas.	“No viable” implica que la estructura parasitaria no puede crecer en el mismo o en un nuevo hospedador intermediario y/o en condiciones de cultivo *in vitro* apropiadas. Sin embargo, la evaluación no invasiva *in vivo* de la viabilidad es todavía imperfecta.
Oncósfera, sustantivo	Forma biológica invasiva de los parásitos *Echinococcus* spp. que surge tras la eclosión del huevo por la acción de enzimas proteolíticas en el sistema digestivo (estómago e intestino) del hospedador intermediario adecuado.	Debe limitarse a la fase que sigue a la ingestión del huevo por parte del hospedador intermediario, justo antes de la fase de proliferación celular (fase post-oncósferal) que constituirá el metacestodo. Véase la figura 1 para descripción detallada y terminología.
Periparasitario, adjetivo	Tejido que rodea al parásito. Para *E. granulosus s.l*.: tejido/ estructuras que rodean la capa laminar de la hidátide. Para *E. multilocularis*: tejido/estructuras que rodean la capa laminar de los microquistes de *E. multilocularis*.	En el quiste desarrollado en la infección por *E. granulosus s.l.* en el hospedador intermediario, la capa adventicia producida por el hospedador, rodeada por el parénquima normal (más los vasos y conductos biliares) representan el tejido “periparasitario”. En las lesiones desarrolladas durante la infección por *E. multilocularis*, el tejido inflamatorio (granuloma) que rodea la lesión sin un límite claro con el parénquima, representa el “infiltrado periparasitario”. En la situación clínica (o animal/experimental) de la EQ, “periparasitario” (incluye la capa adventicia y el parénquima normal del órgano) no debe ser sinónimo de “periquístico” (que sólo incluye el parénquima normal del órgano; véase también la definición de “quiste hidatídico” y la figura 2).
Periquiste, sustantivo periquístico, adjetivo	Tejido que rodea al quiste. En lo que respecta a la equinococosis, el adjetivo se aplica a *E. granulosus sensu lato*, *E. vogeli* y *E. oligarthra*.	El término periquiste sólo corresponde al parénquima del órgano (más los vasos y conductos) que rodea al quiste. No debe utilizarse en la infección por *E. multilocularis* para calificar el tejido/estructura que rodea las lesiones. En la situación clínica (o animal/experimental) de la EQ, el sustantivo “periquiste” (o el adjetivo “periquístico”) no debe utilizarse para designar la capa adventicia.
Post-oncosferal, expresión	Etapa entre la oncósfera y el metacestodo plenamente desarrollado.	Como se refiere textualmente a la “oncósfera”, esta expresión/término se refiere más precisamente a *Echinococcus* spp. que “larva de transición”, que puede aplicarse a cualquier tipo de larva. Desde el punto de vista de la biología e inmunología del desarrollo, las primeras etapas de desarrollo en el hospedador intermediario son cruciales, ya que es el momento en el que el parásito es más susceptible de ser eliminado.
Proglótide o proglótido (singular) proglótides (plural), sustantivo	Parte de la forma adulta de parásitos de *Echinococcus* spp. que se origina a partir del cuello que se halla a continuación del escólex, en el intestino de los hospedadores definitivo.	En referencia al origen griego del término (correa, cuerda), “proglottis (singular), proglottides (plural). En el idioma español ambos vocablos son correctos y equivalentes. En el presente texto utilizamos proglótide y sugerimos éste como de uso corriente. Los proglótides pueden ser inmaduros, maduros o grávidos, según el grado de desarrollo del aparato genital.
Protoescólex (Plural:protoescólex), sustantivo	Forma inicial invaginada del escólex producida por la cápsula prolígera de *Echinococcus* spp. y liberada en el líquido hidatídico.	Del griego antiguo (“scolex”, genitivo: “scolecos” y no “escolicos”) con el prefijo “protos” (antes). Plural invariable en español.
Pseudoquiste, sustantivo pseudoquístico, adjetivo	Entidad anatómica irregular y su imagen característica, similar a un quiste debido a la necrosis central en lesiones de EA en estadios avanzados y EN debida a *E. vogeli.*	Los términos diferencian las imágenes similares a quistes en EA del “quiste” real de EQ (con su estructura parasitaria que incluye las 3 capas). Esta estructura no corresponde a una entidad parasitológica. El término “pseudoquiste” se usa generalmente para las cavidades necróticas que se desarrollaron en el páncreas después de episodios de pancreatitis. Siendo el contexto y el órgano diferente de la pancreatitis y la formación de la cavidad también debido a la necrosis de una lesión inflamatoria, se consideró que el uso de las palabras “pseudoquiste” y “pseudoquístico” también podría usarse en EA.
Quiste hidatídico, expresión	Entidad anatómica producida por el crecimiento del metacestodo de *Echinococcus granulosus s.l.*, distinto del parénquima del órgano que lo rodea y lleno de líquido, que incluye (desde afuera hacia adentro) - Capa adventicia (de origen en el hospedador, incluso reducida a pocas células infiltrantes o a tejido fibroso) - Capa laminar - Capa germinal - Líquido hidatídico (y su contenido)	La palabra “quiste” debe ser reservada a situaciones clínicas (o experimentales) en EQ (quiste hidatídico) o EN; puede ser observado por una variedad de técnicas de imágenes como ultrasonografía, tomografía computada o resonancia magnética. Nunca debe ser usado para designar la cavidad necrótica a menudo desarrollada en las lesiones de EA. “Pseudoquiste” debe ser usado para esta cavidad.
Segmento, sustantivo	Parte de la forma adulta de *Echinococcus* spp. resultante de la proliferación de las células embrionarias del “cuello” del escólex, en el intestino del hospedador definitivo.	En español, equivalente a “proglótide”. Se puede utilizar como equivalente de “proglótide” (para la enseñanza y para el público en general).
Ventosas, sustantivo	Apéndices de la forma adulta de *Echinococcus* spp. que le permite adherirse a la pared intestinal del hospedador definitivo para alimentarse.	También se encuentran presentes en los protoescólex.
Vesícula (parasitaria o equinococócica), sustantivo	Entidad anatómica resultante del crecimiento *in vitro* de *Echinococcus* spp., cualquiera que sea la especie.	Debe reservarse para situaciones *in vitro* donde las estructuras parasitarias del metacestodo no presentan capa adventicia.
Viable (aplicable a metacestodo, capa germinal, quiste, microquiste, protoescólex), adjetivo	Cualquier estructura parasitaria que contiene células vivas capaces de proliferar en condiciones adecuadas.	“Viable” implica que la estructura (independientemente de su tipo) puede crecer en el mismo o en un nuevo hospedador intermediario y/o en condiciones de cultivo *in vitro* apropiadas. Las estructuras parasitarias viables pueden contener o no protoescólex.

Adaptado con autorización de Vuitton DA *et al.* Parasite. 2020;27:41.

**Tabla 3:** Términos en español recomendados para aspectos clínicos de las equinococosis

Palabra/ expresión	Definición	Argumentos para aceptar este término, referencias, aclaraciones lingüísticas, comentarios
Cateterización estándar, Acrónimo en inglés S-Cat	Modificación de la técnica PAIR para el tratamiento de quistes seleccionados, que incluye la introducción de un catéter que puede dejarse o no temporalmente en el quiste.	El nombre del procedimiento y su acrónimo, diferencian a este procedimiento del PAIR convencional y del Mo-CAT. (de Cateterización Modificada). Para completar la descripción se propone añadir: si es en una sesión y el catéter se retira posteriormente, o si son múltiples sesiones siendo el catéter extraído después de otra u otras sesiones. Debe indicarse la forma de guía de la punción percutánea (ultrasonidos, o TAC) así como el tipo y tamaño de los catéteres.
Complicado/a, adjetivo aplicable a quistes de EQ, EN y lesiones de EA	En EQ, EA o EN designa todo incidente espontáneo o provocado (incluyendo los posteriores a intervenciones terapéuticas) que ocurren en los quistes de EQ o EN, o en las lesiones de EA.	Es una definición clínica y las complicaciones pueden ser: rotura (fuera o dentro de las estructuras del órgano infectado); compresión o invasión de estructuras internas del órgano infectado u órganos vecinos; infección bacteriana o fúngica de los quistes en EQ o EN, o de los pseudoquistes en EA; reacciones anafilácticas debido a los antígenos parasitarios (alergia IgE dependiente); compresión o invasión de tejidos o estructuras internas (por ejemplo: en huesos o cerebro) en EQ, EA, y EN. El adjetivo complicado en equinococosis no debe ser utilizado para designar el simple crecimiento del parásito (con o sin dolor) si no tiene consecuencias en las estructuras del órgano afectado u órganos vecinos. El tamaño grande de un quiste y su proximidad a conductos biliares o vasos en el hígado (o bronquios o vasos en el pulmón o estructuras vitales en el cerebro), no son *per se* complicaciones, aunque hagan más difícil o imposible el tratamiento quirúrgico. Son simplemente particularidades anatómicas.
Diseminada, adjetivo (aplicable en EQ, EA y EN)	Forma clínica de la equinococosis quística, alveolar o neotropical diseminada en más de un órgano o tejido.	La definición implica que el quiste o lesión no se limita a un solo órgano. No prejuzga la naturaleza de la diseminación (localización múltiple, invasión local o regional, metástasis, equinococosis secundaria).
Drenaje biliar percutáneo transhepático, acrónimo PTDB (del inglés Percutaneous Transhepatic Biliary Drainage)	Intervención no quirúrgica empleada después de punción percutánea, para el drenaje del árbol biliar en la EA y en la EQ complicada.	No tiene sinónimos. Expresión y acrónimo ampliamente empleados en la literatura, cualquiera sea su aplicación. El procedimiento se aplica tanto en la EQ como en la EA y no implica una acción curativa sobre el quiste hidatídico o sobre las lesiones por EA. Debe indicarse la forma de guía de la punción percutánea (ultrasonido o TAC) así como el tipo y tamaño de los catéteres.
Drenaje biliar perendoscópico, expresión	Técnica no quirúrgica empleada para el drenaje del árbol biliar mediante Colangiopancreatografía retrógrada endoscópica (CPRE en español, ERCP en inglés).	El procedimiento que se aplica tanto en la EQ como en la EA, puede o no incluir la colocación de stents. Esta técnica no implica una acción curativa del quiste hidatídico o de las lesiones por EA; solamente trata las complicaciones de la enfermedad o del tratamiento quirúrgico. En las publicaciones científicas si se asocia con la colocación de stents, debe mencionarse específicamente, así como el número y tipo de stents.
Drenaje percutáneo de la cavidad, expresión	Intervención percutánea transhepática guiada por imagen, utilizada para el drenaje de las cavidades postoperatorias, después de la cirugía por EQ. Se aplica sólo a esta última.	Se considera necesario usar otra expresión para describir el drenaje de una cavidad necrótica en la EA (”pseudoquiste”), diferenciando el tratamiento de la cavidad postoperatoria residual en la EQ. Esta expresión fue añadida en el consenso en idioma inglés para distinguir bien las respectivas situaciones en la EA y la EQ. Debe indicarse el modo de guía de la punción percutánea (por ejemplo: ultrasonido o TAC) así como el tamaño y tipo de cateterización y aspiración.
Drenaje percutáneo de pseudoquiste, expresión	Intervención percutánea transhepática guiada por imagen, utilizada para el drenaje de un pseudoquiste central en lesiones avanzadas de EA. Se aplica solamente a esta última.	En el tratamiento de la EA se prefiere esta expresión a “drenaje de la cavidad”, dado que se refiere al típico pseudoquiste que se forma por necrosis en las lesiones de EA. A su vez, el drenaje de la cavidad se usa habitualmente para el drenaje/tratamiento de las cavidades postoperatorias postquirúrgicas en EQ (quistectomía). Debe indicarse el modo de guía de la punción percutánea (ultrasonido o TAC) así como el tamaño y tipo de cateterización y aspiración.
ELRA, acrónimo en inglés de: *Ex-vivo* Liver Resection with Autotrasplantation (Resección hepática ex vivo con autotrasplante)	Intervención quirúrgica que incluye: hepatectomía total seguida de resección *ex vivo* de las partes patológicas del hígado, asociado con reconstrucción de las vías biliares y/o vasos, seguida por reimplante del hígado.	Técnica quirúrgica empleada en el tratamiento de la EA avanzada, también usada en la EN por *E. vogeli.* Esta expresión debe preferirse a las usadas anteriormente. El acrónimo ha sido propuesto por el equipo quirúrgico que ha realizado la mayoría de dichas intervenciones e incluye todas las etapas de la operación.
Equinococosis, sustantivo (Plural: equinococosis)	Enfermedad o enfermedades producidas por la infección por cestodos del género *Echinococcus*.	Término recomendado por la *World Federation of Parasitologists* (WFP). Siguiendo esta regla la *International Association of Hydatidology modificó su nombre a World Association of Echinococosis* (WAE) en 2015, en el XXVI *World Congress of Echinococcosis*, en Bucarest. Este término puede usarse de manera genérica solamente cuando nos referimos a todas las enfermedades producidas por *Echinococcus* spp. En estudios epidemiológicos y clínicos debe precisarse más la enfermedad distinguiendo entre equinococosis quística producida por *E. granulosus s.l.,* alveolar debida a *E. multilocularis*, y neotropical por *E. vogeli* y *E. oligarthra*.
Equinococosis alveolar, expresión	Enfermedad producida por *E. multilocularis,* con múltiples microquistes dando un aspecto “alveolar” al corte del órgano infectado.	Expresión ampliamente utilizada desde la descripción de la enfermedad en el siglo XIX. Une al nombre del género al adjetivo “alveolar”, genitivo de “alveolaris” (de o relacionado con “alveoli”/ pequeñas cavidades/bolsas de aire en el pulmón). La expresión se ajusta a la recomendación de la WFP (World Federation of Parasitologists) y el adjetivo da precisión a la morfología de la lesión (alveolar) señalando bien el aspecto de las lesiones, especialmente en el hígado. La falta de precisión con respecto al tipo de lesión conduce a malentendidos entre expertos y ejecutores de la línea de acción. Expresión recomendada por la WHO - IWGE desde su primera guía. Diferencia fácilmente la enfermedad debida a *E. multilocularis* de la producida por *E. granulosu*s s.l.
Equinococosis multiquística, expresión	Cualquier tipo de equinococosis con múltiples quistes, observada en el mismo órgano mediante imagen o cirugía.	El término solamente hace referencia a la presencia de múltiples quistes, independientemente de la etiología de la enfermedad.
Equinococosis neotropical, expresión	Enfermedades debidas a *E. oligarthra* y *E. vogeli.*	De acuerdo a las recomendaciones de la WFP el adjetivo neotropical particulariza la enfermedad, evocando la distribución geográfica de las especies de *Echinococcus* involucradas. Aunque la expresión no da ningún dato sobre la morfología de la lesión (“poliquística” en *E. vogeli* y “uniquística” en *E oligarthra*), el adjetivo neotropical distingue la enfermedad causada por esas especies de la EQ y la EA.
Equinococosis quística, expresión	Enfermedad debida a *E. granulosus s.l.*	Expresión de acuerdo con la WFP. Proviene del griego antiguo *“cystis”* (vesícula anatómica) dando precisión a la morfología de la lesión (quística). La falta de precisión en la definición (quística versus alveolar) induce a confusión entre los expertos y ejecutores de los planes de acción. Recomendada por WHO-IWGE desde su primera Guía. Diferencia fácilmente la enfermedad debida a *E. granulosus s.l.* y a *E. multilocularis.*
ERCP, acrónimo en inglés para *Endoscopic Retrograde Cholangio- Pancreatography* (Colangiopancreatografía Retrógrada Endoscópica)	Técnica endoscópica empleada para explorar las vías biliares y pancreáticas, cualquiera sea la enfermedad. Puede o no asociarse con una esfincterostomía u otros procedimientos adicionales.	La realización de una ERCP puede ser no solamente con fines diagnósticos sino también terapéuticos, siendo el primer paso para un drenaje biliar endoscópico. En las publicaciones estas situaciones deben aclararse.
Hepatectomía, sustantivo	Resección del hígado o parte del mismo.	Del griego antiguo *“hepar”,* genitivo “*hepatos*” (hígado), con el sufijo *“ektomé”* / *“ektomia”* (incisión o ablación) que en cirugía se refiere a la extracción de cualquier órgano o lesión, por lo cual literalmente sería la extracción del hígado, aunque la extracción es parcial en la mayoría de las hepatectomías. Están descritas varias técnicas. En las publicaciones científicas siempre debe definirse la técnica quirúrgica empleada según las definiciones usuales en cirugía hepática, indicando los segmentos resecados. Esto es aplicable para todos los tipos de hepatectomía, no solamente en equinococosis.
Hidatidosis, sustantivo	Enfermedad relacionada a infección con *Echinococcus granulosus s.l.*	Nombre comúnmente usado para designar enfermedades debidas a *Echinocccus granulosus s.l.* Si bien este nombre de enfermedad no se ajusta a las recomendaciones de la WFP, en latinoamérica y España existe legislación en medicina humana y veterinaria y en programas de control, que emplea el término para la equinococosis quística. Si bien se recomienda el uso de equinococosis quística como nomenclatura unificada para el futuro, es necesario reconocer que en la práctica la difusión del término hace muy dificultosa su eliminación y reemplazo inmediato. Para el ámbito académico/ científico no debe utilizarse el término Hidatidosis sin incluir equinococosis quística junto con éste o en su reemplazo.
Laparoscopía, sustantivo	Califica cualquier intervención quirúrgica hecha bajo laparoscopía.	Si la intervención es hecha por laparoscopía se sugiere añadir información complementaria.
Laparotomía, sustantivo	Califica cualquier intervención quirúrgica hecha bajo laparotomía.	Intervención quirúrgica que incluye la apertura del abdomen. Debe añadirse al nombre o expresión que describe cualquier tipo de operación si es hecha por laparotomía. Ver Figura 3 y la descripción del sistema AORC con la definición de componentes en cirugía hepática de EQ.
Mo-CAT, acrónimo en inglés para describir Técnica de Cateterización Modificada	Procedimiento percutáneo de tratamiento de la EQ que conduce a la extracción de capas del quiste, incluyendo tal vez vesículas hijas además del líquido hidatídico y los protoescólex.	Técnicas similares a la PEVAC descriptas en otra parte, que no se usan más. El acrónimo Mo-CAT distingue este procedimiento del PAIR convencional y de otras técnicas estándares de cateterización. Debe indicarse el modo de guía de la punción percutánea (por ejemplo: ultrasonido, tomografía computarizada) así como el tipo y tamaño del catéter y de la aspiración.
PAIR, acrónimo inglés de Puncture, Aspiration, Injection of protoescolicide, Reaspiration (Punción, Aspiración, Inyección de protoescolicida, Reaspiración)	Tratamiento percutáneo de la EQ usando punción mediante aguja y protoescolicidas.	Nombre y acrónimo aceptado para designar el procedimiento descrito por Ben Amor *et al.* (11) y publicado en inglés por Gargouri *et al.*^12^. Describe las 4 etapas del procedimiento, el cual no incluye cateterización del quiste. El procedimiento ha sido evaluado y sus indicaciones se han precisado por el WHO-IWGE. El nombre y el acrónimo solamente deben emplearse para el procedimiento inicialmente descrito, empleando punción mediante aguja, aspiración manual con jeringa, sin cateterización u otras técnicas asociadas.
Periquistectomía, sustantivo	Remoción de un quiste EQ que incluye todas las capas del quiste (incluyendo la adventicia).	Usado erróneamente para designar la operación que incluye la capa adventicia del quiste en EQ. El prefijo “peri” no tiene uso, debido a que la capa adventicia es parte del quiste, quistectomía es por esto una palabra apropiada. En sentido estricto el periquiste es el parénquima normal del órgano que rodea el quiste (ver Figura 3) Ver Tabla 3 para vocablos alternativos respecto de la cirugía en EQ. El único sustantivo recomendado es “quistectomía”. Ver también figura 3 de la descripción del sistema AORC con definición de los componentes de la cirugía hepática de EQ.
Protoescolicida, sustantivo	Compuesto natural o químico que es capaz de matar a los protoescólex.	Del griego antiguo scolex, genitivo scolecos; plural scolicos, con el prefijo “protos” y el sufijo latino “cide” (matar). En español las palabras plurisílabas no agudas, no tienen plural: el protoescólex, los protoescólex. Debe utilizarse en lugar de “escolicida”, vocablo de uso muy difundido en publicaciones.
Pseudoquiste, sustantivo Pseudoquística, adjetivo	Entidad anatómica (y en imagen) irregular, en forma de quiste, debida a la necrosis central en las lesiones por EA. No es una entidad parasitológica.	Diferencia el quiste real de EQ (con su estructura parasitaria en 3 capas) de las imágenes en forma de quiste de la EA que simulan un quiste hidatídico, pero no lo son (prefijo pseudo = falso).
Quiste abierto (con Quistectomía total, subtotal y parcial), acrónimo en inglés OC, expresión	Operación quirúrgica que incluye la apertura del quiste antes de extraerlo (quistectomía). Se aplica solamente a la EQ. No se aplica a la EA.	Del punto de vista lingüístico es más correcto que la expresión “quistectomía abierta” dado que un quiste puede o no ser abierto durante la intervención, pero una quistectomía no puede ser abierta o cerrada. La expresión quiste abierto describe bien la situación y el riesgo potencial de diseminación de protoescólex o fragmentos de la capa germinal. Expresión introducida recientemente ( Ver Figura 3).
Quiste hidatídico, expresión	Lesión anatómica producida por la infección por *E. granulosus s. l.,* excluyendo las otras especies de Echinococcus.	Nombre alternativo para designar el o los quistes debidos a la infección por *E. granulosus s.l.* Viene del griego antiguo “cystis” (vesícula). La expresión podría emplearse para casos clínicos (quistes observados en la EQ en humanos u hospedadores intermediarios animales), así como en experimentos in vivo. Esta expresión está de acuerdo con la clasificación internacional de la WHO- IWGE de los quistes observados por ultrasonido (CE1- CE5). No debe emplearse para definir la enfermedad equinococosis quística, dado que existen formas asintomáticas.
Quiste secundario, expresión	Quiste desarrollado a partir de la diseminación de los protoescólex o fragmentos de la capa germinal, en el mismo u otro órgano, de origen espontáneo, accidental o por cirugía.	Modo de formación distinto al de las vesículas hijas. El adjetivo “secundario” se adapta bien a la situación. Asimismo, se ha denominado “hidatidosis secundaria” a la EQ que presenta este tipo de quistes. Esta expresión fue añadida en el consenso original en inglés, para distinguir bien las respectivas situaciones en cuanto origen, diagnóstico y tratamiento de las vesículas hijas y de los quistes secundarios.
Quistectomía, sustantivo	Extracción de un quiste, cualquiera sea su naturaleza, debido a infección por *E. granulosus s.l.*, *E. vogeli* o *E. oligarthra*.	Del griego antiguo “cystis” (vesícula) y “ektomie” (incisión y ablación; cortar y extraer). Literalmente significa extracción del quiste. El contexto debe ser claro para no confundir entre un quiste hidatídico y cualquier otro tipo de quiste. Además, la quistectomía debe ser acompañada por la descripción del tipo de técnica quirúrgica, el tipo de resección y la extensión de la extirpación del quiste. Para esta descripción quirúrgica puede emplearse el sistema AORC: A: tipo de abordaje; O (del inglés open): quiste abierto o quiste no abierto. Se refiere al estado del quiste y el riesgo potencial de diseminación de protoescólex, R: tipo de resección: quistectomía, hepatectomía (lobectomía, segmentectomía); C: Completitud: quiste extraído en su totalidad o no (extracción total, subtotal o parcial).
Quistectomía parcial, expresión	Intervención quirúrgica que solamente extrae partes de las tres capas del quiste hidatídico.	Es lo opuesto a “quistectomía subtotal” la cual solamente deja parte de la capa adventicia en su lugar, pudiendo incluir la remoción parcial de cualquier capa (incluyendo partes de las capas germinal y laminar). Por definición, en este caso, el quiste hidatídico debe ser abierto (en una primera o segunda fase).
Quistectomía periadventicial, adjetivo	Quistectomía total hecha sin abertura del quiste, y en la que se opera por el espacio de disección entre la capa adventicia y el parénquima “normal” del hígado, permitiendo la extracción total del quiste.	El adjetivo indica con precisión que la resección es por fuera de la capa adventicia (la adventicia queda incluida en el quiste resecado). El uso del adjetivo es facultativo.
Quistectomía subtotal, expresión	Quistectomía total con extracción incompleta de la adventicia en un quiste hidatídico.	Situación que sucede cuando limitadas partes del quiste no pueden extraerse con seguridad por la proximidad de vasos sanguíneos u otras estructuras anatómicas (por ejemplo: conductos biliares, bronquios, estructuras cerebrales de función crítica). Para ser calificada como “subtotal” la quistectomía debe extraer la totalidad de la capa germinal y de la capa laminar, dejando solamente partes de la adventicia. Por definición, en esta situación (como en la quistectomía parcial) el quiste ha debido abrirse (en segundo tiempo quirúrgico).
Quistectomía total, expresión	Extracción completa de un quiste hidatídico incluyendo el contenido (líquido hidatídico y los protoescólex) y todas las capas del quiste (germinal, laminar y adventicia).	Ver en la Figura 3 y en la descripción del sistema AORC, la definición de los diferentes componentes de la cirugía de un quiste hidatídico hepático.
Quistectomía total (quiste NO abierto), acrónimo inglés NOC	Intervención quirúrgica que implica la no apertura del quiste antes de extraerlo. Se aplica solamente a la EQ y no es oportuna en EA.	Es más correcto que quistectomía cerrada. Esta denominación describe bien el estado del quiste (no abierto) y el riesgo reducido de diseminación de los protoescólex o de fragmentos de la capa germinal durante la operación. Expresión introducida recientemente (NOC en inglés) que califica con mayor precisión la quistectomía total.
Trasplante de hígado ortotópico, acrónimo en inglés OLT	Extracción del hígado del receptor seguido de trasplante del hígado del donante (o parte del hígado) en el mismo sitio anatómico.	Técnica quirúrgica empleada para el tratamiento de casos avanzados de EA, EN por E. *vogeli* y más raramente de EQ. A veces se usa la expresión “alotrasplante del hígado”. No incluye autotrasplante. El adjetivo ortotópico es facultativo dado que la mayoría de trasplantes de hígado en humanos son ortotópicos.
Tratamiento antiparasitario, expresión	Tratamiento farmacológico de la equinococosis capaz de matar a *Echinococcus* spp. o detener o retrasar su desarrollo, en los diferentes estadios del ciclo del parásito.	Puede aplicarse a los diferentes estadios de la infección por *Echinococcus* spptanto en los hospedadores intermediarios (incluyendo humanos) como en los hospedadores definitivos (mascotas, animales domésticos y silvestres). Las drogas y pautas de tratamiento pueden ser diferentes según los varios estadios y clases de enfermedad.
Vesícula hija. expresión	Hidátide formada dentro del quiste hidatídico (y mucho menos frecuente fuera) durante el desarrollo de *E. granulosus s.l.* Esta estructura parasitaria es claramente distinta de las cápsulas prolígeras que producen los protoescólex. No se aplica a *E. multilocularis*.	Esta expresión ha sido usada durante un siglo y deriva del francés -vésicule fille- propuesta por F. Dévé. Actualmente se acepta que estas estructuras anatómicas derivan de la capa germinal en caso de agresión al metacestodo. Aunque el término “hija” evoca una reproducción sexuada que no existe en el metacestodo, se ha decidido mantener el término por su uso histórico. La vesícula hija no tiene capa adventicia. No debe utilizarse el adjetivo “secundaria” que se refiere a la vesiculización de protoescoléx después de la salida o derrame de un quiste hidatídico roto. El término vesícula se acepta para el cultivo in vitro del metacestodo. (Ver Figura 2).

Adaptado con autorización de Vuitton DA *et al.* Parasite. 2020;27:41.

**Tabla 4:** Términos en español rechazados para la genética y la epidemiología de especies de *Echinococcus.*

Palabra/ expresión	Definición	Argumentos para aceptar este término, referencias, aclaraciones lingüísticas, comentarios
*Alveococcus*, sustantivo	Nombre de género no válido utilizado a menudo en la literatura rusa (en la antigua Unión Soviética en general) para designar a *E. multilocularis* (Leuckart, 1863).	*E. multilocularis* (Leuckart, 1863) pertenece al género *Echinococcus*. Debe aplicarse el Código Internacional de Nomenclatura (ver Tabla 1). Deben utilizarse solo los nombres de especies del género *Echinococcus* mencionados en la Tabla 1. El género *Alveococcus Abuladse*, 1960 (todavía en uso ocasionalmente en la literatura rusa) se utilizó para separar a *E. multilocularis* de las otras especies. Ya no existe una base taxonómica para tal separación.
*Echinococcus alveolaris*, nombre de especie	Nombre de especie inválido a veces utilizado, incluso recientemente, en la literatura alemana y turca para designar la especie *E. multilocularis* o a la enfermedad “equinococosis alveolar” (EA).	Para designar el cestodo, debe aplicarse el código internacional de la nomenclatura zoológica (ver Tabla 1). Para designar a la enfermedad, se seguirán las recomendaciones de La Federación Mundial de Parasitología (WFP) (ver Tabla 3). Debe abandonarse definitivamente.
*Echinococcus cysticu*s, nombre de especie	Nombre de especie inválido utilizado en ocasiones en la literatura alemana y turca para designar a la especie *E. granulosus* o a la enfermedad “equinococosis quística” (EQ).	Para designar el cestodo, debe aplicarse el código internacional de la nomenclatura zoológica (ver Tabla 1). Para designar a la enfermedad, se seguirán las recomendaciones de La Federación Mundial de Parasitología (WFP) (ver Tabla 3). Debe abandonarse definitivamente.
Especies incluidas en *Echinococcus granulosus* sensu lato, (Listado en orden alfabético, no exhaustivo) *E. borealis* *E. cameroni* *E. cepanzoi* *E. intermedius* *E. longimanubrius* *E. lycaontis* *E. minimus* *E. patagonicu*s	Nombres de especies históricos, no válidos dentro del grupo *E. granulosus s.l.*, que han sido posteriormente clasificados como otras especies reconocidas actualmente.	En espera de una definición precisa de especies adicionales dentro de *Echinococcus granulosus s.l.*, se deberían usar los genotipos “G” complementariamente a las “especies” actualmente aceptadas (consulte la Tabla 1). Estos nombres de especies no deben usarse, a menos que se pueda demostrar (p. ej., mediante estudios moleculares) que ameriten el reconocimiento como especies separadas.
*Echinococcus oligarthrus, Echinococcus oligarthus,* nombres de especie	Nombre de la especie dentro del género *Echinococcus* spp. que es responsable de una de las formas clínicas de la equinococosis neotropical.	La especie descrita originalmente por Diesing, *Taenia oligarthra* (Diesing, 1863) es mencionada a menudo por los nombres T. (o E.) *oligarthrus* y T. (o E.) *oligarthus*. Los argumentos históricos y lingüísticos obligan a volver al nombre inicial *“oligarthra”*. Ver Tabla 1. La regla del Código Internacional de Nomenclatura Zoológica es mantener el nombre original de la especie, independientemente de los cambios posteriores que puedan haber ocurrido (incluidos los errores ortográficos).
*Echinococcus sibiricensis,* nombre de especie	Nombre histórico de la especie *E. multilocularis.*	*E. multilocularis* (Leuckart, 1863) es actualmente el único nombre de especie aceptado en la nomenclatura internacional. Posteriormente se demostró que la especie descrita como *E. sibiricensis* (Rausch y Schiller, 1954), era coespecífica con *E. multilocularis* (Leuckart, 1863).

Adaptado con autorización de Vuitton DA *et al.* Parasite. 2020;27:41

**Tabla 5:** Términos en español rechazados relacionado a la biología e inmunología de las especies de *Echinococcus.*

Término/expresión	Concepto o definición	Argumentos para rechazar este término, referencias, aclaraciones lingüísticas, comentarios
Adventicia, sustantivo	Capa fibrosa y celular entre la capa laminada del quiste producido por la infección del *E. granulosus s.l.* y el parénquima normal del órgano del hospedador donde el metacestodo ha desarrollado.	Palabra de origen latino, “adventitia” es el tejido conectivo más externo que cubre un órgano, vaso u otra estructura biológica. En la equinococosis quística (EQ), esta capa pobremente celular y más fibrosa es el resultado entre la respuesta inmune del hospedador contra el metacestodo del *E. granulosus sensu lato.* Como el término “adventicia” podría ser usado para cualquier estructura biológica, se recomienda el uso de “capa adventicia”.
Célula germinal, expresión	Células somáticas pluripotentes con algunas homologías con neoblastos de platelmintos de vida libre. Las células germinativas son las únicas células mitóticamente activas en el metacestodo y dan origen a todas las células diferenciadas	Expresión alternativa para designar las células germinativas. El mismo adjetivo como en la expresión de “capa germinal” donde estas células están localizadas. Sin embargo, no todas las células de la capa germinal corresponden a esta definición. La capa germinal también contiene otro tipo de células tales como células musculares, nerviosas, células productoras de corpúsculos calcáreos.
Células madres somáticas totipotenciales, expresión	Células madre de la capa germinal del metacestodo de *Echinococcus* spp.	Alternativa para “células germinativas”; sin embargo, las células reales totipotentes, con marcadores específicos, no han sido identificadas aún. A la espera de una mejor definición de las células totipotentes de la capa germinal del metacestodo de *Echinococcus* spp, la expresión de “célula germinativa” debería ser mantenida.
Células primarias, expresión	Todas las células que resultan de una digestión lítica del metacestodo del *Echinococcus* spp. y que pueden ser mantenidas usando un “sistema de cultivo de células primarias”.	Célula primaria no es un tipo de célula específica del metacestodo de *Echinococcus* spp. El término se confunde erróneamente con “cultivo primario”, que contiene un conjunto de “células germinativas” (~80% al inicio), células musculares y nerviosas.
Cistoide, adjetivo	Entidad anatómica irregular parecida a un quiste (o su imagen) debido a una necrosis central en lesiones avanzadas de EA así como en la EN (*E. vogeli*).	Deben utilizarse los términos “pseudoquiste” o pseudoquístico” descriptivos en la Tabla 2.
Escólices (como plural de *escólex*), sustantivo	Primer segmento (“cabeza”) de la forma adulta de los cestodos.	Escólex es la forma en plural correcta, por ser palabra de más de una sílaba terminada en x.
Ganchitos, sustantivo	Apéndices del adulto de *Echinococcus* spp. que permiten al parásito adherirse a la pared intestinal del hospedador definitivo.	Este término, un diminutivo de ganchos, también es usado para designar estas estructuras en el escólex de la forma adulta y los protoescólex de *Echinococcus* spp.; debería evitarse su uso porque la denominación de “ganchito” infiere a la versión pequeña de los “ganchos”, es decir que hay dos formas de ellos, los ganchos largos y los pequeños en el órgano de adherencia de algunos parásitos Monogeneos.“Gancho” es el único término recomendado.
Gusano adulto, expresión	Estadio sexual reproductivo de *Echinococcus* spp. en sus hospedadores definitivos.	El término “gusano” se emplea muchas veces como traducción de *“worm”* en inglés. Se puede prestar a confusión con otros organismos como anélidos. A veces se emplea como “estadio adulto”. Gusano adulto se puede considerar como redundante, porque los gusanos son las formas adultas de los helmintos. Adicionalmente, “estadio adulto” en el hospedador definitivo se corresponde con el “estadio larval” en el hospedador intermediario. En trabajos académicos se recomienda la utilización de “helminto”.
Infiltración (periparasítica), sustantivo	Componente histológico (celular y fibroso) originado por el hospedador en la equinococosis alveolar.	Nombre alternativo de “infiltrado”; infiltrado es más común en la terminología patológica; infiltración tiene un significado ligeramente diferente (migración inflamatoria sistemática en todo el órgano).
Larva transicional (o estado), expresión	Estado del *Echinococcus* spp. entre la oncósfera y el desarrollo completo del quiste (i.e. primer estado en el desarrollo del metacestodo).	Expresión alternativa para el estado “postoncosferal”, pero menos preciso para calificar un estado del metacestodo ya que esto se podría aplicar a cualquier larva. Adicionalmente, “transicional” podría calificar cualquier otro estado de desarrollo (por ej. cuando las vesículas germinativas producen protoescólex).
Membrana germinal, expresión	Capa interna celular del metacestodo de *Echinococcus* spp	Este término debe ser evitado y el término “capa” debería ser preferentemente empleado por su complejidad de varias “capas” de la hidátide, y la posible confusión de “membrana” con la membrana celular, la cual tiene una definición específica en biología. Capa germinal es la expresión recomendada para designar la capa interna celular de origen parasitario en el metacestodo de *Echinococcus* spp.
Membrana laminada, expresión	Capa externa acelular del metacestodo de *Echinococcus* spp.	Este término debe ser evitado y la palabra “capa” debería ser empleada debido a la complejidad de varias capas en la hidátide y la posible confusión del uso de “membrana” con la “membrana celular”, la cual tiene una definición específica en biología. Capa laminar es la expresión recomendada para designar la capa externa acelular del parásito originada por el metacestodo de *Echinococcus* spp.
Muerta (lesión quística), adjetivo	Estructura parasitaria no viable evidenciada por imágenes (calcificación completa en EA y CE5 para EQ) o por examen histológico (ausencia de células parasíticas viables).	Este adjetivo suele utilizarse en el contexto clínico o experimental cuando la evidencia de ausencia de viabilidad no es obtenida por transplantes o en cultivos *in vitro*. No se recomienda su uso.
Neoblasto, sustantivo	Tipo de célula madre somática la cual comparte algunas homologías con las células germinativas del metacestodo de *Echinococcus* spp.	Específico de planarias (y otros platelmintos de vida libre). No debería ser empleado para *Echinococcus* spp.
No fértil (forma adulta), expresión	Forma adulta del *Echinococcus* spp. cuyos últimos segmentos no contienen huevos.	Alternativo a “inmaduro” (forma adulta). Sin embargo, no fértil puede inferir que este gusano no es capaz de llegar a ser fértil (lo cual es un error para la mayoría de formas adultas de *Echinococcus* spp, especialmente *in vivo* en el hospedador definitivo). Formas adultas en cultivo in vitro de *Echinococcus* spp. no contienen huevos; éstas presentan algunas diferencias con las formas adultas desarrolladas in vivo, y son posiblemente no fértiles. Sin embargo, la expresión “no fértil” no puede ser aceptada para una situación usual del desarrollo *in vivo* de las formas adultas del *Echinococcus* spp.
Proglotis, sustantivo singular	Relacionado con el origen griego del término: parte del adulto de *Echinococcus* spp. como resultado de la segmentación del escólex en el intestino de los hospedadores definitivos.	Proglótido o proglótide es el término que se recomienda utilizar.
Protoescólices (como plural de protoescólex), sustantivo	Prefijo (“proto”) del escólex, producido por las vesículas germinativas provenientes de la capa germinal del metacéstode de *Echinococcus* spp. y liberados en el fluido del quiste.	Protoescólex es la forma en plural correcta, por ser palabra de más de una sílaba terminada en x.

Adaptado con autorización de Vuitton DA *et al.* Parasite. 2020;27:41

**Tabla 6:** Términos en español rechazados para aspectos clínicos de equinococosis

Término/expresión	Concepto o definición	Argumentos para rechazar este término, referencias, aclaraciones lingüísticas, comentarios
Alveococosis, sustantivo	Enfermedad relacionada a *E. multilocularis.*	Nombre histórico para la infección debida a *E. multilocularis* en Rusia/Ruso y países relacionados con Rusia. El único nombre recomendado es EA. No se usa en otros países o lenguajes fuera del Ruso/Rusia.
Anti-infeccioso, anti- infectivo, adjetivo (Terapia/tratamiento/droga)	Tratamiento con drogas de la equinococosis, asociado o no con la cirugía.	Aunque son usadas comúnmente para el tratamiento de enfermedades infecciosas, estos adjetivos infieren tanto prevención como tratamiento. Antiparasitario es más exacto y más apropiado para enfermedades parasitarias.
*Echinococcus alveolaris*, nombre de especie	Nombre incorrecto relacionado a *E. multilocularis*	El único nombre reconocido es *E. multilocularis,* asociado a la equinococosis alveolar.
*Echinococcus cysticus,* nombre de especie	Nombre incorrecto de especie perteneciente a *E. granulosus s.l.* (*s.l.*)	El único nombre reconocido es *Echinococcus granulosus s.l.*, asociada a la equinococosis quística.
Enfermedad hidatídica, expresión	Enfermedad relacionada a la infección por *Echinococcus* spp.	Comúnmente usado como nombre alternativo para designar todas las enfermedades debidas a *Echinococcus* spp. o las enfermedades debidas a *E. granulosus s.l.* Este nombre de enfermedad no cumple con las recomendaciones unificadas de la Federación Mundial de Parasitólogos. Además, el uso de este nombre incrementa la confusión de clínicos y tomadores de decisión entre enfermedades debidas a *E. granulosus s.l.* y *E. multilocularis* respectivamente. Este sustantivo no se debería usar para echinococcosis alveolar o neotropical, no se recomienda su uso tampoco para infección de *E. granulsosus s.l.* en humanos; el único nombre recomendado para publicaciones académicas es equinococosis quística.
Equinococosis poliquística, expresión	Enfermedad relacionada con la infección por *E. vogeli* (destacando la presentación/tipo multiquística).	La expresión “poliquística” se usa a veces en publicaciones médicas para designar tanto a infecciones producidas por *E. vogeli* que es multiquística como *E. oligarthra* cuya presentación es uniquística. Si bien el término “multiquístico” es el correcto, en países tropicales donde está presente la EN se prefiere el uso de “poliquística” para evitar confusión entre esa enfermedad y la debida a *E. granulosus s.l.* (con presentación de quistes múltiples) y *E. multilocularis*. El adjetivo poliquístico es ampliamente usado para designar una enfermedad genética no parasitaria de hígado y riñón.
Hidático, adjetivo	Relacionado con *Echinococcus* spp.	Galicismo para *“hidátide”,* usado como adjetivo (*‘hydatique’,* en francés), no debe ser usado en español.
Hidátide hija, expresión	Hidátides formadas *de novo* en el interior (y menos frecuentemente fuera, si la hubiera) del quiste de EQ en el desarrollo.	Aunque coincide bien con la descripción de hidátide como sustantivo, no se recomienda su uso. La expresión adecuada es “vesícula hija”.
Hidatidectomía, sustantivo	Quistectomía parcial incluyendo la remoción de capas germinal y laminar del quiste abierto por *E. granulosus s.l.*	Se recomienda el uso de “quistectomía parcial” para referirse a estos procedimientos.
Poliquístico, adjetivo	Constituido de varios quistes (usados para calificar las imágenes y el aspecto operativo de varios tipos de equinococosis).	El adjetivo poliquístico no es específico para equinococosis. Si bien el término “multiquístico” es el correcto, en países tropicales donde está presente la EN por *E. vogeli* se prefiere el uso de “poliquistico” para evitar confusión entre esa enfermedad y la debida a *E. multilocularis* y *E. granulosus* s.l. (con presentación de quistes múltiples). El adjetivo poliquístico es ampliamente usado para designar una enfermedad genética no parasitaria de hígado y riñón.
Poliquistosis hidatídica, expresión	Equinococosis quística (u otra) con quistes múltiples.	Algunas veces usada en publicaciones. Ver poliquístico. El adjetivo multiquístico se propone ahora como descripción no específica de cualquier tipo de equinococosis quística cuando están presentes varios quistes macroscópicos y/o son visibles por imágenes. El uso hidatídico como adjetivo debería ser restringido a infecciones debidas a *E. granulosus s.l.*
Protoscolicida, sustantivo Protoscolicida, adjetivo	Compuesto (natural o químico) que puede matar protoescólex	Sinónimo de protoescolicida. Debe ser abandonado por “protoescolicida” (Ver Tabla 3); protoescolicidas (agentes protoescolicidas) se usan para matar protoescólex y/o prevenir quistes secundarios luego de la cirugía.
Quimioterapia, sustantivo	Tratamiento con drogas de la equinococosis, asociado o no con la cirugía.	Si bien la definición de quimioterapia que se viene usando es correcta para el tratamiento con drogas, independientemente de la enfermedad que se trate, está fuertemente asociada con el tratamiento del cáncer, por lo que se sugiere el uso de tratamiento o terapia antiparasitaria.
Quistectomía abierta, expresión	Operación quirúrgica que incluye apertura de quiste antes de la remoción del mismo (quistectomía)	Muy usado por cirujanos, aunque es lingüísticamente incorrecto: una quistectomía, que es una intervención, siempre es abierta, aunque se abra o no se abra el quiste. Ver también Tabla 3 y figura 3 y la descripción del sistema AORC con la definición de los componentes en la cirugía hepática de EQ. La expresión debe ser reemplazada por quistectomía de quiste abierto (OC)
Quistectomía cerrada, expresión	Operación quirúrgica que no incluye apertura de quiste antes de la remoción del mismo (quistectomía).	Muy usado por cirujanos, aunque es lingüísticamente incorrecto: una quistectomía, que es una intervención, no puede ser cerrada. Ver también Tabla 3 y Figura 3 y la descripción del sistema AORC con la definición de los componentes en la cirugía hepática de EQ. La expresión debe ser reemplazada por quistectomía de quiste no abierto (NOC).
Quistoide, adjetivo	Entidad anatómica parecida a un quiste (o su imagen) debida a necrosis central en lesiones de EA.	Vocablo alternativo para diferenciar las imágenes tipo quiste de la cavidad central necrótica en EA del quiste real de EQ (el sufijo oide que designa algo que se parece a un quiste, pero no lo es). Al contrario de pseudoquiste, quistoide no existe como sustantivo, por lo que debe ser asociado con “cavidad” para designar la estructura.
Scolicida, sustantivo scoleicida, adjetivo	Compuesto (natural o químico) que puede matar protoescolex.	El escolex es la cabeza del adulto de *Echinococcus* spp. El uso de scolicida es por eso científicamente inexacto (matar concierne a protoescolex, no a los scolices) y etimológicamente incorrecto. Debe ser abandonado por protoescolicida

Adaptado con autorización de Vuitton DA *et al.* Parasite. 2020;27:41

## IMPLICANCIAS PRÁCTICAS

El presente trabajo es una traducción y adaptación del primer consenso internacional de términos relacionados con la equinococosis publicado en idioma inglés ^1^, realizado por un grupo de expertos representantes de siete países de Latinoamérica y España. Asimismo, ya ha sido publicada una versión de dicho trabajo en idioma francés ^2^ y chino ^3^, elaborada por expertos de la temática pertenecientes a distintos países francófonos y chino mandarín.

Del mismo modo en que esos trabajos se están empleando como referencia para las publicaciones y difusión científica, proponemos que la presente publicación se incorpore como referencia internacional para el idioma español. Además de incluir terminología para el lenguaje científico y académico, se han considerado vocablos y frases de uso en el lenguaje coloquial.

Las zonas endémicas poseen una enorme extensión geográfica, con muchas poblaciones aisladas y con escaso acceso a la salud de manera frecuente y sistemática. Es importante incluir en la comunicación con los integrantes de estas comunidades, conocimientos y lenguajes que les resulten propios, para poder armonizar la terminología en palabras o frases comunes, de manera progresiva. Pero, sobre todo, resulta indispensable para quienes realizan las tareas de prevención, diagnóstico, vigilancia y control comprender exactamente los significados de términos interculturales asociados a la equinococosis ^10^^-^^12^.

Un ejemplo de la diversidad lingüística existente en la región, son los nombres comunes de los hospedadores definitivos e intermediarios de la EN, importantes de tener en cuenta en la anamnesis de un paciente con sospecha de esta equinococosis, como: *Dasyprocta* spp: agutí, acutí, jochi, sereque, guatín, picure, añuje, cotuza.; *Cuniculus paca*: paca, lapa, majaz, guagua, picuro, jochi pintado, conejo pintado, conejo manchado, tepezcuintle, guartinaja, guanta, tinajo, jaleb, Akutipay, paí.; *Speothos venaticus*: zorro pitoco, zorro vinagre, perro grullero, perro de monte, perro de agua, perro venadero, cachorro do mato vinagre, jagua yvyguy, umba, bush dog; *Dasypus novemcinctus*: armadillo de nueve bandas, mulita grande, tatú jhú, tatú, armadillo, pirca, cusuco, dugu dugu, cusuco.

Las personas que redacten documentos, tanto en el ámbito académico, como en medios donde se emplea el lenguaje coloquial, deben considerar la diversidad terminológica para enfocar la escritura en la manera más clara posible. Es necesario difundir, compartir y sumar organizaciones públicas y privadas, individuos y las ONG a la difusión del presente consenso, con el objetivo de avanzar en el tiempo hacia un lenguaje unificado.

La publicación del consenso en inglés sugiere que el término hidatidosis no debería ser usado para la infección por *E. granulosus sensu lato*, siendo equinococosis quística el nombre recomendado. Sin embargo, en nuestros países tenemos una historia extensa en cuanto a la normativa respecto de estas enfermedades, donde se emplea la denominación de “hidatidosis”. Este término se incluye en reglamentaciones locales, provinciales o estaduales y a nivel país, como así también en programas de control y prevención. Existen también denominaciones en instituciones académicas y sociedades científicas que emplean actualmente el término “hidatidosis”. Por esto, en el presente consenso no se rechaza el término hidatidosis, aunque se sugiere que en el ámbito académico solamente se utilice la denominación de equinococosis. Entendemos que el proceso de unificación de la terminología se debe desarrollar de manera paulatina y sostenida y surgirán en el futuro actualizaciones y revisiones del presente consenso, pudiendo rechazarse la denominación de hidatidosis en el futuro.

Se sugiere que las definiciones de términos, expresiones y recomendaciones proporcionadas en las tablas y las figuras de este trabajo sean seguidas en el futuro para todas las publicaciones y documentos técnicos en español. Asimismo, se sugiere que los editores científicos y los revisores recomienden a los autores que utilicen los términos aprobados.
